# Sequence Type 5 (ST5) as a Possible Predictor of Bacterial Persistence in Adult Patients with Methicillin-Resistant Staphylococcus aureus Pneumonia Treated with Vancomycin

**DOI:** 10.1128/spectrum.01348-22

**Published:** 2022-09-12

**Authors:** Ya-Xin Fan, Meng-Ting Chen, Nan-Yang Li, Xiao-Fen Liu, Min-Jie Yang, Yuan-Cheng Chen, Xiao-Yu Liang, Ju-Fang Wu, Bei-Ning Guo, Si-Chao Song, Yong-Qiang Zhu, Feng-Ying Zhang, Jing-Qing Hang, Sheng-Bin Wu, Bo Shen, Hua-Yin Li, Qin Wang, Xu-Ming Luo, Qing-Ge Chen, Hui-Fang Zhang, Rui-Lan Wang, Li-Hua Shen, Feng-Ming Fu, Xiao-Lian Song, Jing Zhang

**Affiliations:** a Institute of Antibiotics, Huashan Hospital, Fudan University, Shanghai, China; b Key Laboratory of Clinical Pharmacology of Antibiotics, National Population and Family Planning Commission, Shanghai, China; c National Clinical Research Center for Aging and Medicine, Huashan Hospital, Fudan University, Shanghai, China; d Phase I Clinical Research Center, Huashan Hospital, Fudan University, Shanghai, China; e Shanghai-MOST Key Laboratory of Health and Disease Genomics, Chinese National Human, Genome Center at Shanghai, Shanghai, China; f Shanghai Institute for Biomedical and Pharmaceutical Technologies, Shanghai, China; g Department of Pulmonary Medicine, Shanghai Putuo District People's Hospital, Shanghai, China; h Department of Nephrology, Shanghai Ninth People's Hospital, Shanghai, China; i Department of Pulmonary Medicine, Zhongshan Hospital, Fudan University, Shanghai, China; j Department of Respiratory Medicine, Putuo Hospital, Shanghai University of Traditional Chinese Medicine, Shanghai, China; k Emergency & Critical Care Department, Shanghai General Hospital, Shanghai Jiao Tong University School of Medicine, Shanghai, China; l Department of Critical Care, Fudan University Shanghai Cancer Center, Shanghai, China; m Department of Oncology, Shanghai Medical College, Fudan University, Shanghai, China; n Department of Respiratory and Critical Care Medicine, Tenth People's Hospital of Tongji University, Shanghai, China; University of Greifswald

**Keywords:** vancomycin, methicillin-resistant *Staphylococcus aureus pneumonia*, ST5, pharmacokinetic/pharmacodynamic, efficacy

## Abstract

Vancomycin remains the mainstay of treatment for methicillin-resistant Staphylococcus aureus (MRSA) pneumonia. This study assessed risk factors for vancomycin failure in 63 patients with MRSA pneumonia through detailed clinical, microbiological, pharmacokinetic/pharmacodynamic, and genetic analyses of prospective multicenter studies conducted from February 2012 to July 2018. Therapeutic drug monitoring was performed during vancomycin treatment, and the 24-h area under the curve (AUC_0–24_) was calculated. All baseline strains were collected for MIC determination, heterogeneous vancomycin-intermediate S. aureus (hVISA) screening, and biofilm determination. Whole-genome sequencing was performed on the isolates to analyze their molecular typing and virulence and adhesion genes. Clinical signs and symptoms improved in 44 patients (44/63, 69.8%), with vancomycin daily dose (*P = *0.045), peak concentration (*P = *0.020), and *sdrC* (*P = *0.047) being significant factors. Isolates were eradicated in 51 patients (51/63, 81.0%), with vancomycin daily dose (*P = *0.009), cardiovascular disease (*P = *0.043), sequence type 5 (ST5; *P* = 0.017), *tst* (*P = *0.050), and *sec* gene (*P = *0.044) associated with bacteriological failure. Although the AUC_0–24_/MIC was higher in the groups with bacterial eradication, the difference was not statistically significant (*P = *0.108). Multivariate analysis showed that no variables were associated with clinical efficacy; ST5 was a risk factor for bacterial persistence (adjusted odds ratio, 4.449; 95% confidence interval, 1.103 to 17.943; *P = *0.036). ST5 strains had higher frequencies of the hVISA phenotype, biofilm expression, and presence of some adhesion and virulence genes such as *fnbB*, *tst*, and *sec* than non-ST5 strains. Our study suggests that ST5 is a possible predictor of bacterial persistence in MRSA pneumonia treated with vancomycin.

**IMPORTANCE** Few studies have simultaneously examined the influence of clinical characteristics of patients with pneumonia, the vancomycin pharmacokinetic/pharmacodynamic (PK/PD) index, and the phenotypic and genetic characteristics of methicillin-resistant Staphylococcus aureus (MRSA) strains. We assessed risk factors for vancomycin failure in patients with MRSA pneumonia by analyzing these influences in a prospective multicenter study. Sequence type 5 (ST5) was a possible predictor of bacterial persistence in adult patients with MRSA pneumonia (adjusted odds ratio, 4.449). We found that this may be related to ST5 strains having higher levels of vancomycin heterogeneous resistance, biofilms, and the presence of adhesion and virulence genes such as *fnbB*, *tst*, and *sec*.

## INTRODUCTION

Pneumonia, along with other lower respiratory tract infections (LRTIs), is one of the leading causes of morbidity and mortality (1–3). A retrospective analysis conducted in six Latin American countries showed that the incidence of pneumonia ranged from 326.6 to 738.5 per 100,000 inhabitants/year, with an adult inpatient mortality rate of 10.1% to 35.1% (1). In a New York City survey, bacterial infections accounted for 15.2% (6,293/41,400) of all deaths from pneumonia, with Staphylococcus being the second highest-ranking bacterium (4). Methicillin-resistant Staphylococcus aureus (MRSA) is an important drug-resistant organism among staphylococci, accounting for approximately 40.3% of hospital-acquired pneumonia (HAP) caused by S. aureus (5).

Vancomycin is a mainstay agent for the treatment of MRSA infections (6, 7), such as pneumonia, but still has a high rate of treatment failure. In a prospective multicenter clinical study treating adult patients with MRSA pneumonia, clinical and microbiological failure was observed in 36.2% and 24.5% of patients, respectively (8, 9). Factors contributing to vancomycin treatment failure include three factors: patient, drug, and bacterium. Age, comorbidities, intensive care unit (ICU) admission, and severity of disease were clinical factors (10) associated with vancomycin treatment failure. A pharmacokinetic/pharmacodynamic (PK/PD) index of 24-h area under the curve over MIC (AUC_0–24_/MIC) was reported to correlate with the efficacy of vancomycin treatment in MRSA infections (6, 7, 11). In 2020, the international guideline updated the target value of AUC_0–24_/MIC from 400 to a range of 400 to 600 to reduce nephrotoxicity and improve efficacy after vancomycin treatment (6, 7). Lodise et al. (12) prospectively included 265 patients with MRSA bacteremia treated with vancomycin, and day 2 AUC/MIC thresholds were not associated with less treatment failure. According to a prospective study conducted by Huashan Hospital, Fudan University, AUC_0–24_/MIC was not associated with efficacy of vancomycin in Chinese adult patients (9). Other factors, such as the specific phenotypic and genetic characteristics of the strains, have been reported to play an important role in the persistence or efficacy of MRSA infections (13–19). A prospective observational cohort study in 121 cases of MRSA bacteremia showed that sequence type 239 (ST239) was associated with a trend toward decreased mortality with vancomycin treatment (17). The accessory gene regulator (*agr*) operon is a key global regulator that coordinates the control of many key virulence pathways, of which *agr* group II was associated with nonresponse to vancomycin treatment in patients with MRSA infections (18). In a single-center cohort study of heterogeneous vancomycin-intermediate S. aureus (hVISA) pneumonia in MRSA infections treated with vancomycin, linezolid, ceftazidime, and sulfamethoxazole, Panton-Valentine leukocidin (PVL) positivity (adjusted odds ratio [aOR], 6.63; 95% confidence interval [CI], 1.79 to 24.64) and hVISA phenotype (aOR, 3.95; 95% CI, 1.18 to 13.21) were predictors of inpatient mortality (19).

Although many studies have been published on the analysis of risk factors for failure of vancomycin in the treatment of MRSA infections, few studies have simultaneously examined the effects of clinical characteristics of patients with pneumonia, vancomycin PK/PD indices, and phenotypic and genetic characteristics of the MRSA strains. This study aimed to investigate the risk factors for vancomycin treatment failure in patients with MRSA pneumonia by performing detailed clinical, PK/PD, and genetic assessments.

## RESULTS

### Clinical characteristics and efficacy assessment.

A total of 63 Chinese patients with MRSA pneumonia were prospectively enrolled, and their demographic and clinical characteristics are shown in Table 1. The majority of patients were male (69.8%), and the median age was 62 (IQR, 52 to 80) years. Approximately 61.9% patients had a history of ICU admission, and 34.9% had a history of cardiovascular disease. The duration of vancomycin treatment was 12 (IQR, 7 to 15) days, and most patients received combination treatment with β-lactams (61.9%). Of these patients, 44 (69.8%) showed improvement in clinical signs and symptoms after vancomycin treatment and 51 (81.0%) had their bacteria eradicated.

**TABLE 1:** Correlation between demographics, clinical characteristics, and vancomycin treatment outcomes

Characteristic^*a*^	Total (*n* = 63)	Clinical signs and symptoms			Bacterial eradication		
		Improved (*n* = 44)	Not improved (*n* = 19)	*P* value^*b*^	Eradicated (*n* = 51)	Persistence (*n* = 12)	*P* value^*b*^
Demographic							
Age, yr (IQR)	62 (52, 80)	64 (57, 80)	61 (50, 80)	0.459	66 (57, 81)	52 (38, 61)	0.062
Gender, male	44 (69.8)	31 (70.5)	13 (68.4)	>0.999	36 (70.6)	8 (66.7)	>0.999
Wt, kg (IQR)	60 (55, 70)	60 (54, 70)	63 (56, 70)	0.559	60 (54, 70)	65 (57, 71)	0.448
BMI, kg/m^2^ (IQR)	21.5 (19.5, 23.5)	21.5 (19.5, 23.2)	22.1 (20.2, 23.8)	0.519	21.5 (19.5, 23.3)	22.2 (20.3, 24.3)	0.694
ICU							
ICU admission	39 (61.9)	26 (59.1)	13 (68.4)	0.578	33 (64.7)	6 (50)	0.510
ICU stay, days (IQR)	26 (15, 34)	25 (16, 36)	27 (13, 30)	0.903	25 (15, 35)	29 (18, 30)	0.330
Underlying disease/condition							
Cardiovascular disease	22 (34.9)	17 (38.6)	5 (26.3)	0.401	21 (41.2)	1 (8.3)	**0.043**
Diabetes	8 (12.7)	6 (13.6)	2 (10.5)	>0.999	8 (15.7)	0 (0)	0.334
Cerebral apoplexy	14 (22.2)	12 (27.3)	2 (10.5)	0.195	13 (25.5)	1 (8.3)	0.270
Dialysis	2 (3.2)	2 (4.5)	0 (0)	>0.999	2 (3.9)	0 (0)	>0.999
COPD	4 (6.3)	3 (6.8)	1 (5.3)	>0.999	4 (7.8)	0 (0)	>0.999
Trauma	5 (7.9)	3 (6.8)	2 (10.5)	0.633	3 (5.9)	2 (16.7)	0.239
Solid tumor	11 (17.5)	10 (22.7)	1 (5.3)	0.150	10 (19.6)	1 (8.3)	0.674
Autoimmune disease	1 (1.6)	0 (0)	1 (5.3)	0.302	0 (0)	1 (8.3)	0.190
Operation	27 (42.9)	20 (45.5)	7 (36.8)	0.588	21 (41.2)	6 (50)	0.747
Implant							
Venous catheter	45 (71.4)	30 (68.2)	15 (78.9)	0.546	36 (70.6)	9 (75)	>0.999
Endotracheal intubation	24 (38.1)	17 (38.6)	7 (36.8)	>0.999	18 (35.3)	6 (50)	0.510
Tracheotomy	23 (36.5)	16 (36.4)	7 (36.8)	>0.999	17 (33.3)	6 (50)	0.328
Treatment							
Vancomycin daily dose, g/day (IQR)	1.9 (1.4, 2.0)	1.7 (1.4, 2)	2 (1.6, 2)	**0.045**	1.6 (1.3, 2)	2 (2, 2.2)	**0.009**
Vancomycin duration, days (IQR)	12 (7, 15)	12 (8, 14)	11 (7, 15)	0.177	12 (7, 14)	12 (10, 15)	0.830
Combined with β-lactams	39 (61.9)	27 (61.4)	12 (63.2)	>0.999	31 (60.8)	8 (66.7)	>0.999
Combined with rifampin	5 (7.9)	2 (4.5)	3 (15.8)	0.156	3 (5.9)	2 (16.7)	0.239
Combined with quinolones	2 (3.2)	1 (2.3)	1 (5.3)	0.516	1 (2)	1 (8.3)	0.347
Combined with other antibiotics	16 (25.4)	9 (20.5)	7 (36.8)	0.212	12 (23.5)	4 (33.3)	0.481

a BMI, body mass index; ICU, intensive care unit; COPD, chronic obstructive pulmonary disease. Continuous variables are expressed as the median (interquartile range, IQR), and categorical variables are summarized as the number (percentage) of cases.

b *P* values ≤ 0.05 are shown in bold value.

Comparisons of the clinical characteristics between the treatment success and failure groups are shown in Table 1. Vancomycin daily treatment (*P = *0.045) was significantly different between the clinical improvement and nonimprovement groups. Bacteria were more likely to be eradicated in patients with cardiovascular disease (*P* = 0.043) and in those with low vancomycin daily doses (*P* = 0.009).

### Vancomycin therapeutic drug monitoring (TDM) and PK/PD analysis.

The results of vancomycin PK and PK/PD analysis in 63 patients are shown in Table 2. The median trough concentration (*C*_min_), peak concentration (*C*_max_), AUC_0–24_, and AUC_0–24_/MIC were 9.85 mg/L, 24.01 mg/L, 393 mg · h/L, and 467, respectively. All of these indices were close to the target values recommended by the international and Chinese guidelines. Comparisons of the PK and PK/PD indices between the success and failure groups are shown in Table 2. *C*_max_ values were significantly different between the clinical improvement and nonimprovement groups (*P* = 0.020). Although the vancomycin AUC_0–24_/MIC was higher in the eradication group than in the persistence group, the difference was not statistically significant (*P* = 0.108).

**TABLE 2:** Correlation between patient pharmacokinetics, pharmacokinetic/pharmacodynamic indices, and vancomycin treatment outcomes

Characteristic^*a*^	Total (*n* = 63)	Clinical signs and symptoms			Bacterial eradication		
		Improved (*n* = 44)	Not improved (*n* = 19)	*P* value	Eradicated (*n* = 51)	Persistence (*n* = 12)	*P* value
PK^*b*^							
*C*_min_, mg/L	9.85 (5.94, 15.28)	9.75 (5.94, 14.35)	12.38 (6.02, 20.39)	0.365	10.00 (6.19, 15.58)	8.56 (4.72, 14.88)	0.561
*C*_max_, mg/L	24.01 (19.37, 29.64)	23.00 (18.65, 27.12)	25.67 (23.58, 32.96)	**0.020** ^*c*^	23.20 (18.98, 29.64)	25.36 (24.04, 29.46)	0.084
AUC_0–24_, mg · h/L	393 (319, 484)	393 (307, 468)	392 (346, 518)	0.931	396 (306, 511)	377 (350, 461)	0.786
PD							
MIC_50_, mg/L	1	1	1	NA	1	1	NA
MIC_90_, mg/L	1	1	1	NA	1	1	NA
PK/PD							
AUC_0–24_/MIC	467 (336, 674)	467 (320, 643)	491 (358, 748)	0.931	503 (324, 750)	407 (357, 513)	0.108

a PK, pharmacokinetic; PD, pharmacodynamic; PK/PD, pharmacokinetic/pharmacodynamic; *C*_min_, trough concentration; *C*_max_, peak concentration; AUC_0–24_, 24-h area under the curve; MIC_50_, 50% MIC; MIC_90_, 90% MIC; hVISA, heterogeneous vancomycin-intermediate Staphylococcus aureus; AUC_0–24_/MIC, 24-h area under the curve over MIC; continuous variables are expressed as the median (interquartile range).

b All PK parameters were calculated as the weighted average values.

c *P* values ≤ 0.05 are shown in bold value.

### Microbiological and genotypic characteristics.

**FIG 1:**
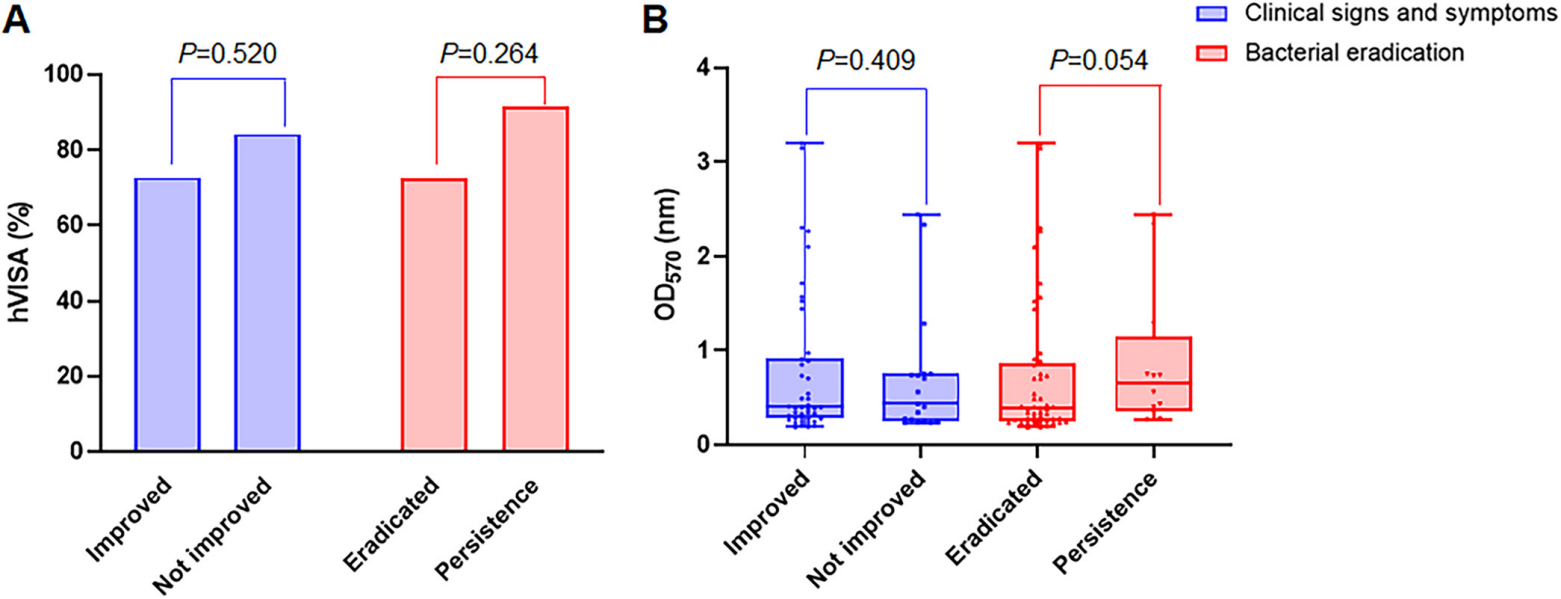
Correlation between heterogeneous vancomycin-intermediate Staphylococcus aureus (A) and biofilm expression (B) of the strains and vancomycin treatment outcomes.

**FIG 2:**
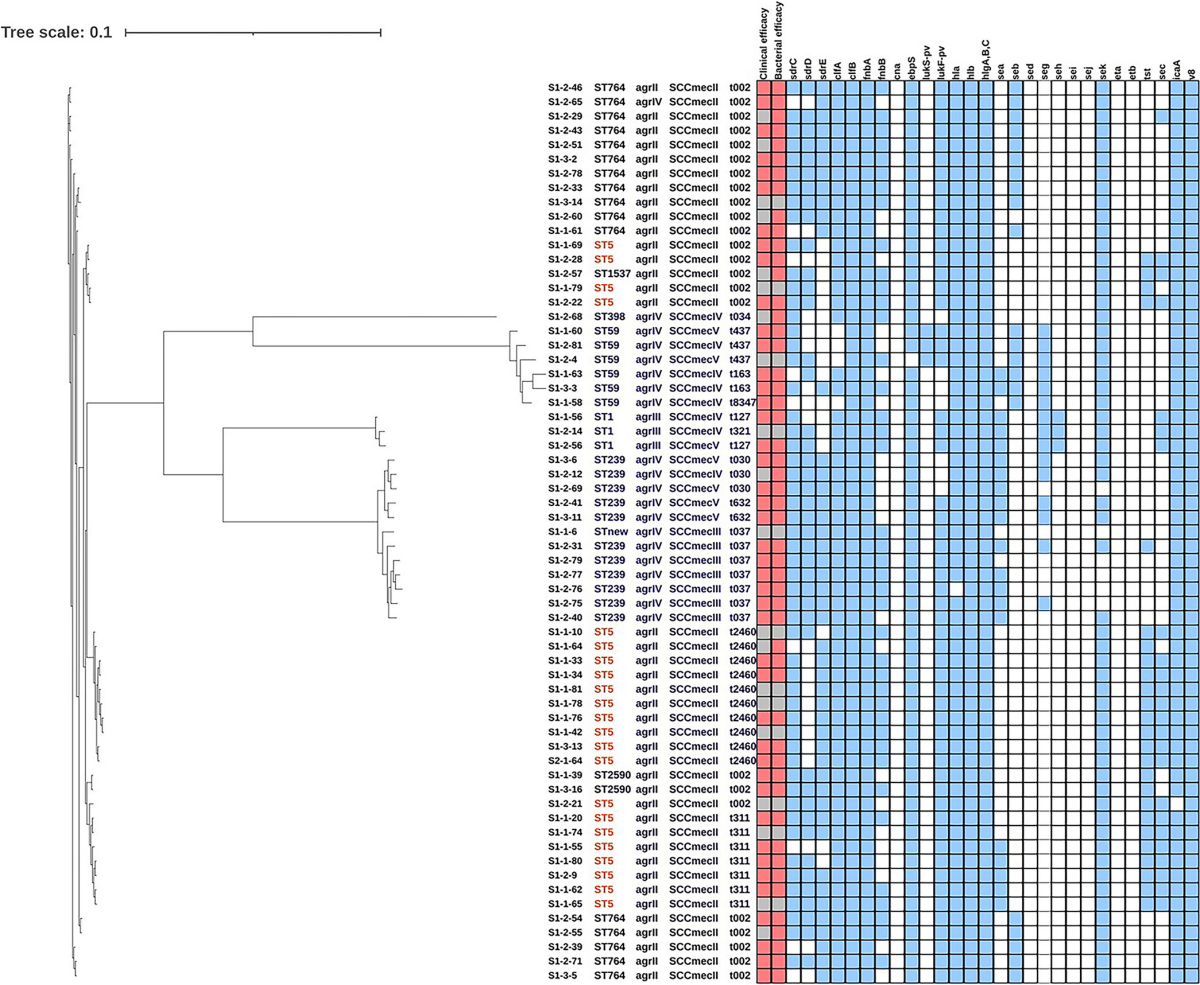
Phylogenetic tree of 63 MRSA strains with the efficacy, molecular typing, and virulence genes. The first column gives the bacterial designation, and columns 2 to 5 show the molecular typing results. In the clinical and microbiological efficacy columns, red indicates treatment success and gray indicates treatment failure. In the virulence factor columns, blue indicates the presence of the gene and white indicates the absence of the gene.

The detection rates of hVISA phenotype and biofilm expression for the two groups are shown in Fig. 1, and a comparison of phenotypes and virulence genes is shown in Table 3. Of these 63 MRSA isolates collected prior to vancomycin treatment, the MIC_50_ and the MIC_90_ for both were 1 mg/L and 48 (76.2%) had the hVISA phenotype. No significant differences in hVISA were observed between the treatment success and failure groups. Biofilm expression was similar in the unimproved and improved groups (0.44 nm versus 0.40 nm, *P = *0.409), and although the bacterial persistence group had higher biofilm expression, the difference was not statistically significant (0.65 nm versus 0.39 nm, *P = *0.054). The phylogenetic tree of the bacteria is represented in Fig. 2. The common ST genotypes were ST5 (34.9%), ST764 (25.4%), and ST239 (17.5%). The major staphylococcal cassette chromosome *mec* element (SCC*mec*), *agr*, and staphylococcal protein A (*spa*) types were SCC*mec* II (65.1%), *agr*II (63.5%), and *t002* (38.1%), respectively. As shown in Table 3, the ST5 genotype was associated with bacterial eradication (*P = *0.017). The virulence factors *tst* (*P* = 0.050) and *sec* (*P* = 0.044) were significantly associated with bacterial eradication, and presence of the *sdrC* gene was significantly different between the clinical success and failure groups (*P = *0.047).

**TABLE 3:** Correlation analysis of molecular typing and virulence factors of pathogenic bacteria with vancomycin treatment outcomes

Characteristic^*a*^	Total (*n* = 63)	Clinical signs and symptoms				Bacterial eradication		
		Improved (*n* = 44)	Not improved (*n* = 19)	*P* value^*b*^		Eradicated (*n* = 51)	Persistence (*n* = 12)	*P* value^*b*^
Molecular typing								
ST5	22 (34.9)	14 (31.8)	8 (42.1)	0.566		14 (27.5)	8 (66.7)	**0.017**
SCC*mec* II	41 (65.1)	27 (61.4)	14 (73.7)	0.401		32 (62.7)	9 (75.0)	0.516
*agr*II	40 (63.5)	26 (59.1)	14 (73.7)	0.394		31 (60.8)	9 (75.0)	0.510
*spa*-t002	24 (38.1)	16 (36.4)	8 (42.1)	0.779		21 (41.2)	3 (25.0)	0.345
Adhesion and virulence factors								
*clfA*	59 (93.7)	41 (93.2)	18 (94.7)	>0.999		48 (94.1)	11 (91.7)	>0.999
*clfB*	63 (100)	44 (100)	19 (100)	NA		51 (100)	12 (100)	NA
*fnbA*	63 (100)	44 (100)	19 (100)	NA		51 (100)	12 (100)	NA
*fnbB*	40 (63.5)	25 (56.8)	15 (78.9)	0.153		30 (58.8)	10 (83.3)	0.183
*sdrC*	54 (85.7)	35 (79.5)	19 (100)	**0.047**		42 (82.4)	12 (100)	0.187
*sdrD*	42 (66.7)	27 (61.4)	15 (78.9)	0.247		33 (64.7)	9 (75)	0.735
*sdrE*	46 (73.0)	33 (75.0)	13 (68.4)	0.758		38 (74.5)	8 (66.7)	0.719
*cna*				NA				NA
*ebpS*	62 (98.4)	44 (100)	18 (94.7)	0.302		51 (100)	11 (91.7)	0.190
*lukS*	3 (4.8)	2 (4.5)	1 (5.3)	>0.999		2 (3.9)	1 (8.3)	0.476
*lukF*	56 (88.9)	39 (88.6)	17 (89.5)	>0.999		44 (86.3)	12 (100)	0.329
*hla*	62 (98.4)	43 (97.7)	19 (100)	>0.999		50 (98)	12 (100)	>0.999
*hlb*	63 (100)	44 (100)	19 (100)	NA		44 (100)	19 (100)	NA
*hlgA*	63 (100)	44 (100)	19 (100)	NA		44 (100)	19 (100)	NA
*hlgB*	63 (100)	44 (100)	19 (100)	NA		44 (100)	19 (100)	NA
*hlgC*	63 (100)	44 (100)	19 (100)	NA		44 (100)	19 (100)	NA
*tst*	25 (39.7)	16 (36.4)	9 (47.4)	0.575		17 (33.3)	8 (66.7)	**0.050**
*sea*	21 (33.3)	18 (40.9)	3 (15.8)	0.080		19 (37.3)	2 (16.7)	0.307
*seb*	19 (30.2)	15 (34.1)	4 (21.1)	0.379		17 (33.3)	2 (16.7)	0.318
*sec*	24 (38.1)	14 (31.8)	10 (52.6)	0.160		16 (31.4)	8 (66.7)	**0.044**
*sed*				NA				NA
*seg*	16 (25.4)	13 (29.5)	3 (15.8)	0.350		14 (27.5)	2 (16.7)	0.714
*seh*	3 (4.8)	2 (4.5)	1 (5.3)	>0.999		2 (3.9)	1 (8.3)	0.476
*sei*				NA				NA
*sej*				NA				NA
*sek*	57 (90.5)	40 (90.9)	17 (89.5)	>0.999		46 (90.2)	11 (91.7)	>0.999
*eta*				NA				NA
*etb*				NA				NA
*icaA*	62 (98.4)	44 (100)	18 (94.7)	0.302		51 (100)	11 (91.7)	0.190
*v8*	63 (100)	44 (100)	19 (100)	NA		44 (100)	19 (100)	NA

a ST, sequence type; SCC*mec*, staphylococcal cassette chromosome *mec*; *agr*, accessory gene regulator; *spa*, staphylococcal protein A; NA, not applicable. All variables are summarized as the number (percentage) of cases.

b *P* values ≤ 0.05 are shown in bold value.

### Risk factors for the clinical and microbiological failure.

Risk factors were identified by logistic regression models with significant univariate variables in Tables 1 to 3. Multivariate analysis (Table 4) showed that only *sdrC* was included in the equation of clinical efficacy, but there was no statistical difference associated with the presence of *sdrC* gene (*P = *0.999). ST5 and vancomycin daily dose were the variables included in the equation associated with bacterial persistence, and ST5 was an independent risk factor (adjusted odds ratio [aOR], 4.449; 95% confidence interval [CI], 1.103 to 17.943, *P* = 0.036).

**TABLE 4:** Multivariable logistic regression for risk factors associated with vancomycin treatment failure in patients with MRSA pneumonia

Risk factor^*a*^	Univariate analysis result [OR (95% CI)]	Multivariate analysis result	
		aOR (95% CI)	*P* value^*b*^
Clinical signs and symptoms			
Vancomycin daily dose	1.757 (0.633, 4.882)		
Peak concn	1.013 (0.979, 1.047)		
*sdrC*	8.770E8 (0.000, ∞)	8.770E8 (0.000, ∞)	0.999
Bacterial eradication			
Vancomycin daily dose	4.332 (1.137, 16.506)	3.744 (0.906, 15.468)	0.068
Cardiovascular disease	0.130 (0.016, 1.084)		
**ST5**	**5.286 (1.372, 20.361)**	**4.449 (1.103, 17.943)**	**0.036**
*tst*	4.000 (1.054, 15.184)		
*sec*	4.375 (1.148, 16.676)		

a ST, sequence type; OR, odds ratio; CI, confidence interval; aOR, adjusted odds ratio.

b *P* values ≤ 0.05 are shown in bold value.

A comparison of the phenotypes of ST5 and non-ST5 strains is shown in Table 5. The hVISA detection rate for ST5 strains was significantly higher than that of the non-ST5 group (100% versus 65.9%, *P* = 0.001). All ST5 strains were SCC*mec* II, compared to only 46.3% of the non-ST5 group. The *agr* type of all ST5 strains is II, while the predominant *agr* type of the non-ST5 strains is IV. ST5 strains had higher biofilm expression than non-ST5 strains (0.71 nm versus 0.34 nm, *P = *0.016). In addition, the presence of *fnbB*, *tst*, and *sec* genes was also significantly higher in ST5 strains than in non-ST5 strains (*P < *0.05).

**TABLE 5:** Comparison of phenotypes of ST5 and non-ST5 strains

Characteristic^*a*^	Value for strains		*P* value^*c*^
	ST5 (*n* = 22)	Non-ST5 (*n* = 41)	
Bacterial phenotype			
hVISA	22 (100)	27 (65.9)	**0.001**
Biofilm, nm (IQR)	0.71 (0.39, 1.57)	0.34 (0.25, 0.74)	**0.016**
Molecular typing			
SCC*mec* II	22 (100)	19 (46.3)	**<0.001**
*agr*II	22 (100)	18 (43.9)	**<0.001**
*spa*-t002	5 (22.7)	19 (46.3)	0.102
Adhesion and virulence^*b*^ factors			
*clfA*	21 (95.5)	38 (92.7)	>0.999
*fnbB*	19 (86.4)	21 (51.2)	**0.007**
*sdrC*	21 (95.5)	33 (80.5)	0.144
*sdrD*	18 (81.8)	24 (58.5)	0.093
*sdrE*	16 (72.7)	30 (73.2)	>0.999
*ebpS*	21 (95.5)	41 (100)	0.349
*lukS*	1 (4.5)	2 (4.9)	>0.999
*lukF*	21 (95.5)	35 (85.4)	0.405
*hla*	21 (95.5)	41 (100)	0.349
*tst*	21 (95.5)	4 (9.8)	**<0.001**
*sea*	7 (31.8)	14 (34.1)	>0.999
*seb*	6 (27.3)	13 (31.7)	0.780
*sec*	19 (86.4)	5 (12.2)	**<0.001**
*seg*	4 (18.2)	11 (26.8)	0.544
*seh*	0 (0)	3 (7.3)	0.546
*sek*	19 (86.4)	38 (92.7)	0.413
*icaA*	22 (100)	40 (97.6)	>0.999

a hVISA, heterogeneous vancomycin intermediate *S. aureus*; ST, sequence type; SCCmec, staphylococcal cassette chromosome mec; agr, accessory gene regulator; spa, staphylococcal protein A. Continuous variables are expressed as the median (interquartile range), and categorical variables are summarized as the number (percentage) of cases.

b The *clfB*, *fnbA*, *hlb*, *hlgA*, *hlgB*, *hlgC*, and *v8* genes were 100% in both groups.

c *P* values ≤ 0.05 are shown in bold value.

## DISCUSSION

In this study, ST5 was a possible predictor of bacterial persistence in patients with MRSA pneumonia after vancomycin treatment. ST5 MRSA was a predominant hospital-associated MRSA (HA-MRSA) clone in East China (20, 21), and the treatment failure of HA-MRSA was higher than that of community-acquired MRSA (22). ST5 was the prevalent sequence type of hVISA, probably because all ST5 stains were SCC*mec* II from nosocomial infections, while 15 (36.6%) strains in the non-ST5 group were of the SCC*mec* IV and SCC*mec* V types, both of which were predominantly derived from community-acquired infections. In addition, biofilm expression was higher in ST5 than non-ST5 strains, although there was no statistical difference between the two groups in terms of success and failure of vancomycin treatment. The high expression of biofilms in ST5 strains compared to non-ST5 strains may be related to the presence of *fnbB* (86.4% versus 51.2%, *P = *0.007). This is consistent with the finding of Cha et al. (23), i.e., that MRSA strains that formed biofilms had a higher frequency of *fnbB* genes than nonforming ones (74.4% versus 45.0%, *P = *0.001). The higher rate of *agr*II in ST5 strains than in non-ST5 strains may also be related to the bacterial persistence of ST5 strains. It has been shown that *agr* group II predominates in glycopeptide intermediately resistant S. aureus isolates (24). The study of Moise-Broder et al. (18) included 87 patients with MRSA infection, and there was a significant difference agrII polymorphism between the vancomycin treatment failure and success groups (69% versus 28%, *P = *0.003). In terms of virulence factors, ST5 strains also had a high prevalence of *tst* and *sec* genes, which were significant factors associated with microbiological efficacy in the univariate analysis. *tst* and *sec* genes encode toxic shock syndrome toxin 1 (TSST-1) and staphylococcal enterotoxin C of the S. aureus superantigen (SAg) family, respectively (25). SAgs can cause staphylococcal toxic shock syndrome, with symptoms including fever, rash, desquamation, hypotension, and hemodynamic shock, thus compromising the effectiveness of treatment (25). ST5 strains had higher vancomycin heterogeneous resistance, biofilm expression, and presence of adhesion and virulence genes such as *fnbB*, *tst*, and *sec* than non-ST5 strains, which may lead to bacterial persistence of vancomycin against MRSA infection.

As we know, AUC_0–24_/MIC is the best PK/PD index related to the efficacy of vancomycin in the treatment of Gram-positive infections (6, 7, 11). An AUC_0–24_/MIC of ≥400 was first reported as a vancomycin PK/PD target for S. aureus pneumonia (26). However, in this prospective multicenter study of adult patients with MRSA pneumonia, higher vancomycin exposure indices, including AUC_0–24_/MIC, AUC_0–24_, and trough and peak concentrations, were not associated with lower treatment failure rates. This is similar to that reported in the literature, where a prospective multicenter (*n* = 14) study of 265 hospitalized patients with MRSA bacteremia tested an AUC_0–24_/MIC target of 400 and found no association with treatment failure (12). In addition, vancomycin penetrates most tissues but in varying concentrations, especially in the lungs (27). Cruciani et al. (28) analyzed a mean penetration rate of 0.41 in lung tissues, and although vancomycin lung tissue concentrations correlated well with serum concentrations (*r* = 0.81), the penetration rate was variable among patients (0.10 to 1.12). This variation may be an important reason for the failure of the AUC_0–24_/MIC of the serum to correlate significantly with vancomycin efficacy.

This study has several limitations. First, the number of cases included was small, and vancomycin concentrations in epithelial cells of lung tissue were not collected. In addition, several other factors, including the minimal bactericidal concentration and *agr* function, were not investigated. Finally, these data have not been validated *in vitro* or in animal models, and further validation of the relevance to bacterial persistence is required.

In conclusion, a high AUC_0–24_/MIC index was not associated with improved clinical signs and symptoms or bacterial eradication. ST5 was a possible predictor of bacterial persistence in adult patients diagnosed with MRSA pneumonia, which may be related to ST5 strains having higher levels of vancomycin heterogeneous resistance, biofilm, and the presence of adhesion and virulence genes such as *fnbB*, *tst*, and *sec*. Further studies are needed to validate these findings.

## MATERIALS AND METHODS

### Study design.

This study was performed using data from two multicenter, prospective observational studies with adult patients (age, ≥18 years) from 8 teaching hospitals in China from February 2012 to July 2018. Adult patients who had clinical symptoms, signs, laboratory tests, pulmonary imaging and microbiology culture for the diagnosis of MRSA pneumonia, requirement for TDM, and a ≥5-day vancomycin treatment period were included in the data set. Patients were excluded if they had received any other anti-Gram-positive agents for ≥24 h within 72 h prior to enrollment and were colonized with Gram-positive bacteria, had received dialysis treatment, were pregnant or lactating women, or were taking concomitant aminoglycosides and polymyxins. The studies were approved by the Ethics Committee of Huashan Hospital and registered with the China Clinical Trial Register (ChiCTR-OPC-16007920 and ChiCTR-OPC-17012567).

### Clinical data and efficacy assessment.

Data on demographics, ICU admission, underlying disease, implant, treatment, history of hospitalization, laboratory tests, pulmonary imaging, bacteriological examination, and vancomycin administration were collected. The clinical effectiveness of vancomycin treatment included both improvement in clinical signs and symptoms and eradication of the bacteria. Improvement in clinical symptoms and signs was defined as the patient’s clinical symptoms and signs of infection, return of laboratory test results (except for bacteriological examinations) to normal or preinfection status, and vancomycin being no longer required within 7 days of drug withdrawal; if there was no improvement, the clinical treatment was ineffective. Eradication of bacteria was defined as the inability to culture the original pathogen within 7 days of the end of vancomycin treatment and the absence of the need for antibiotics against Gram-positive organisms. Persistence of bacteria was defined as the ability to culture the original pathogen from the infection site or/and a lack of improvement in clinical symptoms, signs, and laboratory test results after the vancomycin treatment in cases where respiratory specimens were not available.

### TDM of vancomycin and PK/PD analysis.

Serum trough concentration samples were collected 0 to 0.5 h before the fifth dose, and peak concentration samples were collected 0.5 to 1 h after the fifth dose. For patients with a creatinine clearance rate (CL_CR_) below 30 mL/L, serum samples were collected at the second dose. Vancomycin concentration was measured by the chemiluminescence microparticle immunoassay or fluorescence polarization immunoassay method. The linear range of vancomycin was 3 to 100 mg/L.

A one-compartment model (Phoenix WinNonlin 8.0 software, Pharsight, USA) was used to simulate the concentration-time of each patient after vancomycin treatment. Matlab 7.0 software (MathWorks, England) was used to calculate AUC_0–24_ for each patient at each dose. For patients administered vancomycin for more than 24 h, the mean corrected AUC_0–24_ was used. The weighted average AUC_0–24_ (WAUC_0–24_) for all doses was calculated based on the duration of treatment, and AUC_0–24,_ *_i_* was calculated for each dose. Also, the weighted average *C*_min_ (W*C*_min_) and *C*_max_ (W*C*_max_) were also calculated as the AUC_0–24_. AUC_0–24,_ *_i_*, *C*_min, i_ and *C*_max,_ *_i_* were the AUC_0–24_, *C*_min_, and *C*_max_ obtained at the *i*^th^ dose, and *D_i_* was the treatment duration of the *i*^th^ dose (days).

The formulas for the calculation were as follows:

$$\mathrm{WAU}C_{0–24}=\frac{\sum_{i}^{k}AUC_{0–24,\;i}\times D_{i}}{\sum_{i}^{k}D_{i}}$$

$$WC_{\min}=\frac{\sum_{i}^{k}C_{\text{min},\;i}\times D_{i}}{\sum_{i}^{k}D_{i}}$$

$$WC_{\max}=\frac{\sum_{i}^{k}C_{\text{max},\;i}\times D_{i}}{\sum_{i}^{k}D_{i}}$$

where *i* = 1, 2, …, *k.*

### Microbiological data and whole-genome sequencing.

Clinical isolates of S. aureus were collected from patients' deep sputum specimens and identified using a VITEK 2 Compact. These sputum specimens were collected from each patient prior to treatment with vancomycin, and the qualifying criteria were ≤10 squamous epithelial cells at low magnification and ≥25 leukocytes. Oxacillin and vancomycin MICs were verified by an agar dilution method in a CHINET microbiology laboratory. The standards for MIC testing were Clinical and Laboratory Standards Institute (CLSI) M100-S31 (29) documents. Strains with an oxacillin MIC of ≥4 mg/L were defined as MRSA. hVISA was confirmed by a modified population analysis profile area-under-the-curve (PAP-AUC) method, as described previously (30). Isolates were standardized in tryptone soy broth (TSB) at a turbidity of 0.5 McFarland for 24 h, and then the cultures were diluted in saline to 10^−1^, 10^−3^, and 10^−6^. Cultures and dilutions (original bacterial solution, 10^−1^, 10^−3^, and 10^−6^) were plated on freshly prepared brain heart infusion agar (BHIA) plates containing 0.5, 1, 2, 2.5, 4, and 8 mg/L vancomycin. All plates were incubated at 35°C for 48 h. Colonies for each vancomycin concentration were counted and plotted in log_10_ CFU/mL by GraphPad Prism 8 (GraphPad Software, CA, USA) software. AUC was calculated for each test isolate and compared to that of a reference strain (Mu3; ATCC 700698). The resulting AUC ratio was classified according to the following criteria: non-hVISA (<0.9), hVISA (0.9 to 1.3), and vancomycin-intermediate S. aureus (VISA; ≥1.3). Biofilm formation was determined by a crystalline violet method and controlled with the ATCC 29213 standard strain. Fresh bacteria grown overnight were selected, adjusted for turbidity to 0.5 McFarland, and shaken at 180 rpm overnight at 37°C. Bacterial broth was diluted 1:100 and inoculated into BHI plus 1% glucose broth after addition to 96-well Costa plates, making 3 replicate wells per strain. After incubation at 37°C for 24 h, the wells were washed three times with phosphate-buffered saline (PBS; pH 7.2). Methanol was used to stabilize the biofilm, followed by 15 min of staining with 1% crystal violet dye. The wells were washed with slow-flowing water until the water was colorless and then dried at room temperature. The contents of each well were dissolved by adding 0.2 mL of an aqueous 80% ethanol solution, and the optical density (OD) was measured at 570 nm on a spectrophotometer (model ELX800; BioTek, Winooski, VT).

The MRSA strains were cultured overnight, and a single colony was picked up and inoculated with TSB at 35°C for 16 to 20 h. After centrifugation, the supernatant was discarded, and the genomic DNA was extracted using the TaKaRa MiniBEST bacterial genomic DNA extraction kit (Ver.3.0) (TaKaRa Biomedical Technology Co., Ltd., Beijing, China). The concentration and purity of DNA were checked using a NanoDrop 2000 ultra-micro spectrophotometer (Thermo Fisher Scientific, Waltham, MA, USA), followed by sequencing using a HiSeq X10 second-generation whole-genome sequencer (Illumina, CA, USA).

Sequence data extracted for seven housekeeping genes (*arcC*, *aroE*, *glpF*, *gmk*, *pta*, *tpi*, and *yqiL*) of S. aureus were used for multilocus sequence typing (MLST), and the ST clone type of each strain was confirmed according to the PubMLST database (https://pubmlst.org/). SCC*mec* types were identified using specific gene fragments of SCC*mec* I to V, as described by Oliveira et al. (31) and Hanssen et al. (32): type I (*pls*; GenBank accession no. AF115379.2), type II (*kdpC*^+^ *kdpB*^+^ *kdpE*; GenBank accession no. BA000018.3), type III (*pI258*; GenBank accession no. GQ900378.1), type V (*hsdM^+^ hsdS^+^ hsdR*; GenBank accession no. AB781450.1), and type IV for the remaining strains. To distinguish between *agr* types I to IV, the *agr* gene of each strain was compared to the following four genes in the NCBI GenBank database: *agr*I (GenBank accession no. AF210055), *agr*II (GenBank accession no. AF001782), *agr*III (GenBank accession no. AF001783), and *agr*IV (GenBank accession no. AF288215). BLAST software was used to find the similarity and length. Each *spa* gene was extracted from the genome, and the strain was assigned to the *spa* type according to the methods in the Ridom SpaServer database (https://www.spaserver.ridom.de/). The genomes of strains were screened for S. aureus virulence genes using the Virulence Factor Database (VFDB; http://www.mgc.ac.cn/VFs/main.htm). The presence of 30 virulence genes, including adhesion and toxin genes, was assessed in the database.

ST5 (GenBank accession no. BA000017.4), ST239 (GenBank accession no. CP002643.1), ST59 (GenBank accession no. CP020741.1), and ST1 (GenBank accession no. NZ_CP019574.1) sequences were selected as reference sequences. The fastq files were compared with the reference sequences to obtain single nucleotide polymorphisms (SNPs) for 63 clinical strains, and the bacterial SNPs were cross-tabulated using MAFFT and MEGA-X 10.0.4 software (Mega Limited, Auckland, New Zealand) to plot gene evolution trees. The phylogenetic tree was integrated with molecular typing, vancomycin treatment efficacy, and virulence factors using iTOL v5 (https://itol.embl.de/).

### Statistical analysis.

All statistical analyses were performed using SPSS 19 (SAS Institute, Inc., Cary, NC, USA). Continuous variables were compared using a Mann-Whitney U test, and categorical variables were compared using the chi-square test or Fisher’s exact test. Continuous variables were expressed as the median (interquartile range [IQR]), and categorical variables were summarized as the number of cases (percentages) in descriptive statistics. A *P* value ≤ 0.05 was considered significant. Multivariate logistic regression was performed using backward stepwise logistic regression to determine variables that were independently associated with clinical and bacterial efficacy.

### Data availability.

The complete genome sequences and annotation have been deposited in the National Center for Biotechnology Information database (https://www.ncbi.nlm.nih.gov/bioproject/) under accession number PRJNA860943 (see the supplemental material). All genomic database data are also available at https://www.ncbi.nlm.nih.gov/biosample/ under accession numbers SAMN29924237 to SAMN29924299.
